# Determination of pK_A_ of nonvolatile weak acids in plasma of healthy volunteers and critically ill patients

**DOI:** 10.1186/s40635-025-00762-8

**Published:** 2025-06-02

**Authors:** Martin Krbec, Serena Brusatori, Petr Waldauf, Alberto Zanella, Francesco Zadek, Victor van Bochove, František Duška, Thomas Langer, Paul Elbers

**Affiliations:** 1 Department of Anaesthesia and Intensive Care Medicine, Third Faculty of Medicine, Charles University and FNKV University Hospital, Prague, Czech Republic; 2 Fondazione IRCCS Ca’ Granda Ospedale Maggiore Policlinico, Milan, Italy; 3 Department of Pathophysiology and Transplantation, University of Milan, Milan, Italy; 4 Department of Medicine and Surgery, University of Milano-Bicocca, Monza, Italy; 5 Department of Intensive Care Medicine, Amsterdam Medical Data Science (AMDS), Amsterdam Cardiovascular Science (ACS), Amsterdam Institute for Infection and Immunity (AII), Amsterdam Public Health, Amsterdam UMC, Vrije Universiteit, Amsterdam, The Netherlands; 6 Department of Anesthesia and Intensive Care Medicine, Niguarda Ca’ Granda, Milan, Italy

**Keywords:** Acid–base equilibrium, Models, Theoretical, Acids, Serum albumin, Hydrogen-ion concentration

## Abstract

**Background:**

The dissociation constant of nonvolatile weak acids in plasma (K_A_), expressed as pK_A_, is essential for electroneutrality-based acid–base analysis. To date, its normal value in human plasma has been determined in only one study involving eight healthy volunteers. We hypothesized that pK_A_ would differ in ICU patients, whose plasma protein composition is altered by disease and medication, and that changes in protein charge—rather than undetected strong acids—could account for the unexplained anions observed in sepsis.

**Methods:**

Using CO_2_ tonometry, we determined pK_A_ and total weak nonvolatile acids (A_TOT_) in plasma from 30 healthy volunteers and two ICU cohorts (27 postoperative and 30 septic patients). Additionally, we calculated the strong ion gap in plasma and protein-free serum filtrates from 10 healthy volunteers and 20 septic patients.

**Results:**

In healthy volunteers, pK_A_ was 7.55 ± 0.16 (K_A_ = 2.8 × 10⁻⁸) and A_TOT_ was 15.9 ± 3.0 mmol/L (0.222 ± 0.043 mmol/g of TP). In postoperative and septic patients, A_TOT_ was significantly reduced (10.1 ± 5.4 and 11.9 ± 4.0 mmol/L, p < 0.001), but pK_A_ and A_TOT_/TP remained unchanged, yielding an average pK_A_ of 7.55 ± 0.35 (K_A_ = 2.8 × 10⁻⁸) and A_TOT_/TP of 0.230 ± 0.097 mmol/g. We found elevated strong ion gap in both plasma and protein-free filtrates of septic patients, which confirms the presence of unmeasured low-molecular-weight anions.

**Conclusion:**

Our findings confirm stable pK_A_ and A_TOT_/TP values in human plasma in both health and disease, supporting the Staempfli-Constable model for clinical acid–base diagnostics. Unexplained anions in sepsis are attributed to low molecular weight strong ions rather than alterations in plasma protein dissociation.

**Supplementary Information:**

The online version contains supplementary material available at 10.1186/s40635-025-00762-8.

## New and noteworthy

We determined the dissociation constant (pK_A_) of weak nonvolatile acids in plasma for both healthy volunteers and critically ill patients with and without sepsis. We hypothesized that pK_A_ values would differ significantly in ICU patients and that shifts in protein charges could account for the high strong ion gap commonly observed in sepsis. Contrary to our expectations, the pK_A_ values were consistent across different groups. These findings suggest that a single, universal pK_A_ of 7.55 is applicable for acid–base calculations in both healthy and critically ill patients and that the elevation of strong ion gap in sepsis is due to low molecular weight anions.

**Supplementary Information:**

The online version contains supplementary material available at 10.1186/s40635-025-00762-8.

## Introduction

Stewart’s physico-chemical model of acid–base equilibrium [1–3] is an eye-opening concept for understanding the biochemical mechanisms affecting the acid–base equilibrium in plasma. In this model, three independent variables determine its acid–base status. Strong ion difference (SID) represents the difference in charge between strong cations and strong anions. These ions are always fully dissociated, and their charge is, therefore, not altered by acid–base processes. Partial pressure of carbon dioxide (PCO_2_) characterizes the effect of carbonic acid species. Finally, nonvolatile weak acids, whose charge varies with pH, are represented by a hypothetical monoprotic acid (HA ⇌ H^+^ + A^−^) with known concentration (A_TOT_ = [HA] + [A^−^]) and dissociation constant (K_A_ or its negative decadic logarithm pK_A_).

In Stewart’s original text, [A^−^] is used as a proxy for the total amount of charge carried by plasma proteins and small molecules such as phosphate. However, later research has indicated that for clinical application such assumption may be oversimplistic, as part of the charge carried by the proteins is pH-independent [4–7] and, as such, complies with Stewart’s definition of strong ions. To reflect this, the true SID of plasma can be considered as the sum of the SID of *measured* strong ions (e.g., Na^+^, Cl^−^, L-lactate), the SID of *unmeasured* strong ions (e.g., β-hydroxybutyrate, oxalate, D-lactate), and the *fixed*, pH-independent portion of protein and phosphate charge:

1 $$SID={SID}_{measured}+{SID}_{unmeasured}+{Pr}_{fix}^{-}+{Phos}_{fix}^{-}$$

With these premises, the hypothetical acid HA as well as its characteristics (A_TOT_ and pK_A_) only refer to the titratable, pH-dependent portion of protein and phosphate charge. If not stated otherwise, this terminology is used throughout the article.

The value of pK_A_ and the relation between A_TOT_ and total protein (TP) or albumin concentration are poorly documented [7, 8] and their experimental determination in humans has only been reported once in a population of 8 healthy volunteers by Staempfli and Constable [6]. In their study, PCO_2_ was manipulated through tonometry and repeated measurements of the relevant parameters (pH, PCO_2_, and electrolyte concentrations) were performed to estimate pK_A_ and A_TOT_ by nonlinear regression, providing K_A_ of 0.8 × 10^–7^ (pK_A_ = 7.1) and A_TOT_ of 17.2 mmol/L (equivalent to 0.224 mmol/g of protein or 0.378 mmol/g of albumin). Additionally, the amount of strong charge attributable to protein was derived: Pr^−^_fix_ = 3.7 mEq/L (equivalent to 0.052 mEq/g of protein or 0.090 mEq/g of albumin).

The values published by Staempfli and Constable have only been validated by the original authors [6]. In addition, whether pK_A_ and A_TOT_ remain consistent under various disease states has not been determined. Yet, critical illness, and especially sepsis, is strongly linked to oxidative stress [9] and oxidation of the albumin molecule is known to alter its properties [10], including buffer power [11]. Furthermore, exogenous albumin, in which the proportion of oxidized molecules is high [12], is often administered to patients in the intensive care unit (ICU). It could be hypothesized that the alterations in the composition and/or function of plasma proteins during critical illness would result in changes of either the pK_A_, the relation between A_TOT_ and TP or albumin concentration, or the strong charge that they exhibit (Pr^−^_fix_). This would introduce bias into acid–base interpretation, including the determination of circulating unmeasured ions (SID_unmeasured_). Indeed, several studies [13–18] have reported an increased strong ion gap (SIG) not explained by lactate in patients with septic shock, but the responsible anions have never been reliably identified. An alteration in the charge of plasma proteins could explain this discrepancy.

In this study, we aimed to evaluate pK_A_ and A_TOT_ in a group of healthy volunteers using contemporary equipment, comparing the results with previous estimates and with findings from two distinct populations of ICU patients—those with and without sepsis (Experiment A). We hypothesized that critical illness and/or sepsis might alter the acid–base properties of weak nonvolatile acids. Additionally, to investigate potential alterations in the fixed charge of plasma proteins (Pr^−^_fix_), we conducted a series of experiments in which the strong ion gap (SIG) was measured simultaneously in plasma and in protein-free serum filtrates obtained from a separate cohort of healthy volunteers and septic patients (Experiment B).

## Materials and methods

### Study overview

This observational prospective case–control study was conducted at two centers: Fondazione IRCCS Ca’ Granda Ospedale Maggiore Policlinico, Milan, Italy and FNKV University Hospital, Prague, Czechia between March 2019 and June 2023.

The study consisted of two parts: SID, pK_A_, and A_TOT_ determination by CO_2_ tonometry of plasma (Experiment A) and quantification of unmeasured ions in protein-free serum filtrates (Experiment B). *Experiment A* included three study groups: (1) Healthy volunteers, (2) patients admitted to the ICU after major elective surgery without signs of infection (Postoperative patients), and (3) patients with sepsis or septic shock as per Sepsis-3 criteria [19] admitted to the ICU (Septic patients). *Experiment B* included two study groups: (1) Healthy volunteers, and (2) patients with sepsis or septic shock admitted to the ICU, using the same inclusion criteria as for Experiment A.

For both experiments, we excluded patients with conditions that could alter the composition or function of plasma proteins or interfere with their concentration measurements. Exclusion criteria included pregnancy, plasma total bilirubin > 4 mg/dL, thalassemia, transfusion of more than four units of red blood cells and/or 1 L of plasma within 24 h before enrollment, liver cirrhosis, and hematological malignancies.

The study was conducted in accordance with the Declaration of Helsinki, approved by the ethics committee at both centers and registered at ClinicalTrials.gov (NCT03966664, registration date 27th May 2019). Written informed consent was obtained according to local regulations.

### Procedures and measuremens

In ICU patients, blood samples were taken preferably from a central venous line. If it had not been placed or if the sample could not be easily drawn, an arterial line was used instead. In healthy volunteers, the blood was taken by standard venipuncture from a peripheral vein on the upper extremity.

In *Experiment A*, the blood was collected in a syringe with dry electrolyte-balanced heparin (safePICO Aspirator, Radiometer, Denmark) for baseline blood-gas analysis, a clot activator tube for biochemistry examination in serum (magnesium, phosphate, TP, and albumin), and several lithium heparin tubes for obtaining plasma by centrifugation (1000 g, 12 min). PCO_2_ of the plasma samples was then manipulated in the range of 15 to 120 mmHg at 37 °C using a CO_2_ tonometer (EQUILibrator, RNA Medical, Devens, MA, USA) and humidified custom-made gas mixtures (0%, 2%, 12% and 20% CO_2_ in air by Linde, Ireland) at a rate of 80 ± 15 mL/minute. During the tonometry, pH, PCO_2_ and electrolyte concentrations were repeatedly measured using a blood gas analyzer (ABL90 FLEX PLUS, Radiometer, Denmark), collecting at least 20 datapoints for each sample.

In *Experiment B,* we collected samples that allowed us to estimate SIG in a way consistent with routine clinical practice (SIG_plasma_): a syringe with dry electrolyte-balanced heparin for immediate blood-gas analysis (providing pH, PCO_2_, [Na^+^], [K^+^], [Ca^2+^], [Cl^−^], and [Lac^−^]) and a clot activator tube for serum biochemistry tests (magnesium, phosphate, TP, and albumin). Additionally, two clot activator tubes were collected to obtain serum, which was then deproteinized by centrifugation (4000 g, 15 min) in dedicated filter units with nominal molecular weight limit of 10 kDa (Amicon Ultra-15, Merck, Darmstadt, Germany). In the protein-free filtrates, the same set of biochemistry tests was repeated, allowing us to calculate SIG_filtrate_.

### Calculations

For Experiment A, the simplified electroneutrality equation [20]

2 $$SID-\left[{HCO}_{3}^{-}\right]-\left[{A}^{-}\right]=0$$

was expanded with the Henderson-Hasselbach equation for [HCO_3_^−^], and Stewart’s equations for [A^−^] [1], providing:

3 $$SID - S \times PCO_{2} \times 10^{{pH - pK_{1}^{\prime } }} - \frac{{A_{TOT} }}{{1 + 10^{{pK_{A} - pH}} }} = 0$$

where S (0.0307 mmol/L/mmHg) represents CO_2_ solubility coefficient and pK_1_’ (6.105) is the apparent first dissociation constant of carbonic acid in isolated plasma [21]. Equation 3 was then used by a nonlinear mixed-effects model to estimate SID, pK_A_, and A_TOT_ in each subject, as first described by Constable [20].

In each subject, the estimate of A_TOT_ was normalized for albumin (A_TOT_/Alb) and TP concentration (A_TOT_/TP), allowing for comparison between individuals with different protein concentrations. [A^−^] at the pH of 7.4 ([A^−^]_7.4_) was calculated using the [A^−^] term in Eq. 3 and individual pK_A_ and A_TOT_ values, illustrating the amount of pH-dependent charge carried by nonvolatile weak acids. Finally, we focused on the difference between the estimated SID and SID_measured_, which represents the net charge of unmeasured strong ions along with the fixed charge of plasma proteins and phosphate (see Eq. 1). To do so, we defined:

4 $${SID}_{measured}=[{Na}^{+}]+[{K}^{+}]+2\times [{Ca}^{2+}]+2\times [{Mg}^{2+}]-[{Cl}^{-}]-[{Lac}^{-}]$$

5 $$\Delta SID=SID-mean({SID}_{measured})$$

where mean(SID_measured_) stands for the mean SID_measured_ from all blood-gas analyses in each plasma sample. The use of the mean value minimizes random and systemic analytical errors [22] but neglects the effect of pH-related ion–protein binding [23–26].

In *Experiment B,* SIG was calculated in plasma and in protein-free filtrates of serum using the standard Figge’s formula [5, 27]:

6 $$SIG = \left[ {Na^{ + } } \right] + \left[ {K^{ + } } \right] + 2 \times \left[ {Ca^{2 + } } \right] + 2 \times \left[ {Mg^{2 + } } \right] - \left[ {Cl^{ - } } \right] - \left[ {Lac^{ - } } \right] - S \times PCO_{2} \times 10^{{pH - pK_{1}^{\prime } }} - Alb \times \left( {0.123 \times pH - 0.631} \right) - Pi \times \left( {0.309 \times pH - 0.469} \right)$$

where pK_1_’ = 6.095 for blood-gas analyses performed in whole blood [21]. In protein-free serum filtrates, we used pK_1_’ = 6.105, which is the value recommended for isolated plasma [21]. This approach is justified by our analysis (Figure S1 and Text S1 in the Supplementary Material), which confirmed the applicability of this value both in isolated plasma and protein-poor fluids.

### Statistics

The sample size for the primary endpoint of Experiment A was calculated using the software SigmaPlot 11.2 (Systat Software Inc., San Jose, CA) with unpaired t-test, focusing on the difference in K_A_ of isolated plasma between critically ill patients with sepsis and healthy controls as the outcome parameter. Based on previous studies [6, 28], we considered a difference in K_A_ of 1.9 × 10^–7^ as clinically relevant and estimated a standard deviation of 2.2 × 10^–7^. After defining the following parameters: minimum detectable difference in means = 1.9 × 10^–7^, expected standard deviation of residuals = 2.2 × 10^–7^, desired power = 0.90, and alpha error = 0.05, the estimated sample size was n = 30 for each group. Given the lack of preliminary data about postoperative patients, we pragmatically decided to aim for the same sample size.

Data analysis was conducted using R version 4.4.1 [29] with the RStudio graphical interface. The normality of the distribution of continuous data was tested using the Shapiro–Wilk test. Continuous parameters that met the assumption of normality (p > 0.05, Shapiro–Wilk test) are reported as mean ± standard deviation (SD); otherwise, they are presented as median (25th–75th percentile). Categorical data are presented as count (percentage).

The relationship between normally distributed continuous parameters was analyzed using Pearson’s correlation coefficient and linear regression. Differences in normally distributed continuous parameters were analyzed using an unpaired t-test for two groups (Experiment B) and one-way ANOVA for three groups (Experiment A). If the data were not normally distributed, the Wilcoxon rank sum test was used for two groups, and the Kruskal–Wallis test for three groups. Categorical data were compared using the chi-square test or Fisher’s exact test, with the latter applied when expected cell frequencies were less than 5.

A nonlinear mixed-effects (NLME) model was employed to estimate plasma acid–base parameters (SID, pK_A_, and A_TOT_) using the nlme 3.1–166 package [30]. The model included fixed effects for experimental groups (healthy volunteers, postoperative patients, septic patients) and random intercepts to account for subject-specific variability within groups. The regression model was specified according to Eq. 3. Model parameters were estimated simultaneously using the maximum likelihood approach. Initial parameter values were determined through exploratory data analysis and iteratively refined during model fitting. To enhance convergence, control parameters, such as the maximum number of iterations and tolerance levels, were optimized. From the NLME model, individual values for SID, A_TOT_, and pK_A_ were calculated. These values were further used to compute A_TOT_/Alb, A_TOT_/TP, [A^−^]_7.4_, and ΔSID. Differences between groups were analyzed using the aforementioned parametric or nonparametric tests as appropriate. No imputation was performed for missing data. A p-value < 0.05 was considered the threshold for statistical significance.

## Results

We enrolled 30 healthy volunteers, 27 postoperative patients, and 30 septic patients for Experiment A. Additional 10 healthy volunteers and 20 septic patients participated in Experiment B. Table 1 presents the baseline characteristics of all studied subjects.

**Table 1:** Baseline subject characteristics in Experiments A and B

	Experiment A			Experiment B	
	Healthy volunteers N = 30	Postoperative patients N = 27	Septic patients N = 30	Healthy volunteers N = 10	Septic patients N = 20
Site, n (%) in Prague	14 (47)	17 (63)	17 (57)	10 (100)	20 (100)
Sex, n (%) of F	14 (47)	9 (33)	12 (40)	5 (50)	9 (45)
Age, years	54 ± 15	61 ± 16	56 ± 18	31 ± 7*	64 ± 16*
Time since ICU admission, days	–	1 (1–2)^#^	2 (1–3)^#^	–	2 (1–5)
Time since sepsis diagnosis, days	–	-	2 (1–3)	–	1 (1–2)
SOFA	–	4.4 ± 1.4^#^	9.7 ± 2.5^#^	–	11.1 ± 3.9
Fluid balance since ICU admission, L	–	4.0 (0.1–6.3)	5.1 (2.0–9.1)	–	5.0 (2.5–10.9)
Mechanical ventilation, n (%)	–	1 (4)^#^	26 (87)^#^	–	15 (75)
ECMO, n (%)	–	0 (0)	5 (16.7)	–	0 (0)
Vasopressor administration, n (%)	–	7 (26)^#^	26 (87)^#^	–	17 (85)
ICU mortality, n (%)	–	1 (4)	7 (23)	–	7 (35)
Total protein, g/L	72 ± 5*°	50 ± 6*^#^	47 ± 7°^#^	76 ± 2*	48 ± 7*
Albumin, g/L	48 ± 3*°	32 ± 4*^#1^	24 ± 4°^#^	48 ± 2*	26 ± 5*
Magnesium, mmol/L	0.86 ± 0.07	0.83 ± 0.12	0.92 ± 0.21	0.82 ± 0.04	0.90 ± 0.15
Phosphate, mmol/L	1.1 ± 0.1	1.0 ± 0.3^#^	1.2 ± 0.6^#^	1.2 ± 0.2	1.5 ± 0.9
pH	7.38 ± 0.03^2^	7.40 ± 0.04^#3^	7.35 ± 0.09^#^	7.34 ± 0.04	7.36 ± 0.12
pCO_2_, mmHg	49 ± 6°^2^	45 ± 5^3^	43 ± 9°	55 ± 9*	44 ± 8*
[HCO_3_^−^], mmol/L	28.6 ± 2.1°^2^	27.5 ± 3.3^#3^	24.5 ± 6.8°^#^	29.2 ± 2.1*	25.2 ± 5.5*
SBE, mmol/L	3.4 ± 1.9°^2^	2.6 ± 3.8^#3^	− 1.1 ± 7.9°^#^	3.5 ± 1.7	− 0.3 ± 7.0
[Na^+^], mmol/L	142 ± 2^2^	139 ± 3^3^	142 ± 6	144 ± 2	141 ± 7
[K^+^], mmol/L	4.2 ± 0.4^2^	3.9 ± 0.4^3^	4.1 ± 0.6	4.3 ± 0.4	4.5 ± 0.8
[Ca^2+^], mmol/L	1.21 ± 0.03*°^2^	1.11 ± 0.08*^3^	1.11 ± 0.10°	1.25 ± 0.02*	1.09 ± 0.14*
[Cl^−^], mmol/L	105 ± 2^2^	105 ± 3^3^	107 ± 6	104 ± 2	106 ± 5
[Lac^−^], mmol/L	1.0 ± 0.5°^2^	1.5 ± 2.3^3^	2.9 ± 3.8°	1.5 ± 0.8	2.3 ± 2.5
SID_measured_, mEq/L	43.7 ± 2.4*°^2^	39.4 ± 2.8*^3^	39.1 ± 6.2°	46.5 ± 2.6*	40.4 ± 5.4*
β-hydroxybutyrate, mmol/L	0.1 (0.05–0.3)*°^4^	0.4 (0.1–0.7)*^5^	0.4 (0.2–0.4)°^6^	N/A	0.2 (0.06–0.4)^7^

Data are presented as mean ± SD, median (1Q–3Q), or N (%) as appropriate. For β-hydroxybutyrate, values below the detection limit (0.1 mmol/L) were treated as 0.05. Superscripts (*, °, and ^#^) denote significant differences in between-group comparisons (p or adjusted p < 0.05). ^1^N = 26, ^2^N = 28, ^3^N = 24, ^4^N = 27, ^5^N = 20, ^6^N = 24, ^7^N = 12 (missing data)

**Fig. 1:**
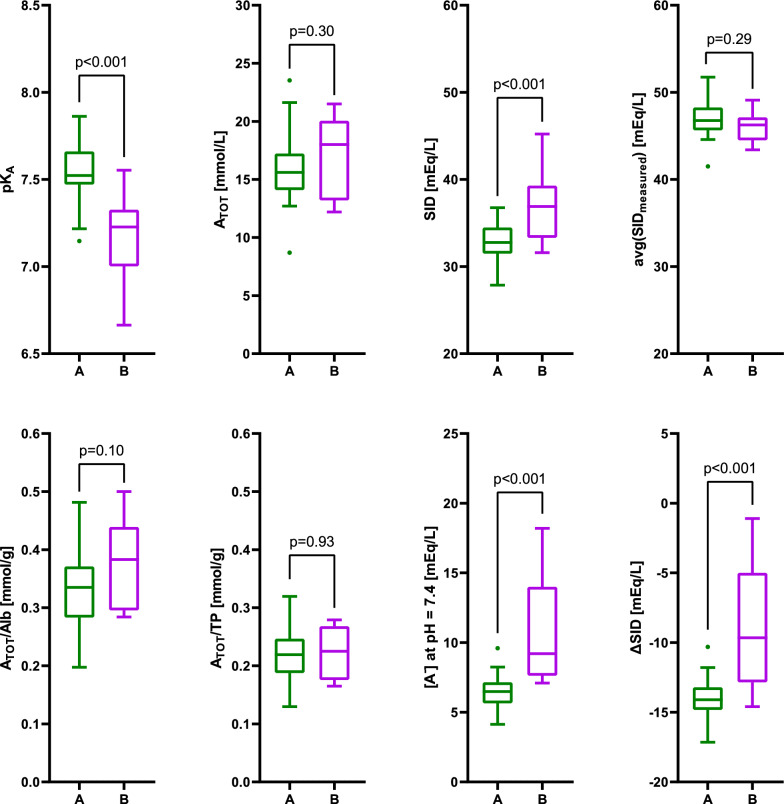
Experiment A. Comparison of the results obtained in healthy volunteers in this study (A, green) and by Staempfli and Constable (B, purple). First row: the primary parameters (pK_A_, A_TOT_, and SID) determined by the nonlinear mixed effects model (our study) or nonlinear regression (Staempfli and Constable) and the average SID due to measured electrolytes (Na^+^, K^+^, Ca^2+^, Mg^2+^, Cl^−^, and Lac^−^). Second row: modifications of the primary parameters (A_TOT_ expressed per gram of albumin or TP, [A^−^]_7.4_, and the difference between the estimated and average measured SID (ΔSID) in each plasma sample

The results of determination of pK_A_, A_TOT_ and SID in the three groups enrolled in Experiment A are shown in Table 2. The results obtained in our cohort of healthy volunteers are contrasted with the values published by Staempfli and Constable [6] in Fig. 1.

**Table 2:** Experiment A

	Healthy volunteers N = 30	Postoperative patients N = 27	Septic patients N = 30	p value
pK_A_	7.55 ± 0.16	7.60 ± 0.44	7.51 ± 0.40	0.6
A_TOT_, mmol/L	15.9 ± 3.0*°	10.1 ± 5.4*	11.9 ± 4.9°	< 0.001
SID, mEq/L	32.9 ± 1.9*°	28.9 ± 2.8*	26.7 ± 5.9°	< 0.001
A_TOT_/Alb, mmol/g	0.333 ± 0.064°	0.318 ± 0.180^#§^	0.505 ± 0.244°^#^	< 0.001
A_TOT_/TP, mmol/g	0.222 ± 0.043	0.204 ± 0.111	0.261 ± 0.116	0.07
[A^−^]_7.4_, mEq/L	6.5 ± 1.1*°	3.4 ± 1.8*^#^	4.7 ± 1.7°^#^	< 0.001
ΔSID, mEq/L	− 14.0 ± 1.4	− 13.6 ± 2.6	− 15.5 ± 3.5	0.02

Estimates of the parameters characterizing weak nonvolatile acids (pK_A_ and A_TOT_) and strong ions (SID) in plasma of the three studied populations. Subsequent analyses focused on the relationship between A_TOT_ and measured plasma albumin and total protein concentration (A_TOT_/TP and A_TOT_/Alb), the charge of weak nonvolatile acids at the pH of 7.4 ([A^−^]_7.4_), and the difference between the estimated SID and SID_measured_ (ΔSID). The p value refers to one-way ANOVA, with superscripts (*, °, and ^#^) denoting significant differences (adjusted p < 0.05) in between-group comparisons by Student’s t-test. ^§^N = 26 (missing data)

Analysis of the results from healthy volunteers, postoperative patients, and septic patients revealed significant differences in estimated A_TOT_ and SID, but not in pK_A_ and A_TOT_/TP. For the latter two parameters, results from pooled data across all participants were also calculated, yielding a pK_A_ of 7.55 ± 0.35 (K_A_ = 2.8 × 10⁻⁸) and an A_TOT_/TP of 0.230 ± 0.097 mmol/g.

**Fig. 2:**
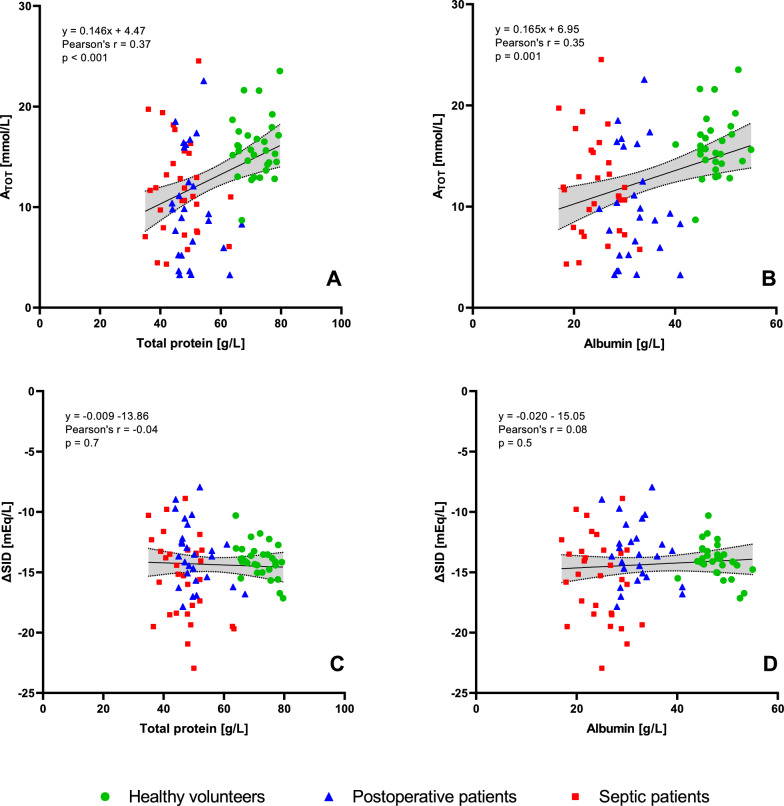
Experiment A. Top panels: the relationship between the estimated A_TOT_ and TP or albumin concentration. Bottom panels: the relationship between ΔSID (i.e., the fixed charge of plasma proteins and phosphate, and unmeasured strong ions) and TP or albumin concentration. Linear regression line with 95% confidence bands is shown in all graphs

While TP and albumin concentration were strongly correlated (Pearson’s r = 0.93, p < 0.001, Figure S2 in the Supplementary Material), A_TOT_ showed only weak correlations with TP (Pearson’s r = 0.37, p < 0.001, Fig. 2A), and albumin (Pearson’s r = 0.35, p = 0.001, Fig. 2B) in pooled data from all participants. Regression analyses of A_TOT_ versus TP and albumin within each group are shown in Figures S3A and S3B in the Supplementary Material.

ΔSID, the parameter representing the fixed protein and phosphate charge along with the charge of unmeasured strong ions, did not differ between healthy volunteers and either patient population. Moreover, no correlation was observed between ΔSID and TP (Pearson’s r = − 0.04, p = 0.7, Fig. 2C) or albumin (Pearson’s r = 0.08, p = 0.5, Fig. 2D) in pooled data from all participants. Regression analyses of ΔSID versus TP and albumin within each group are shown in Figures S3C and S3D in the Supplementary Material.

**Fig. 3:**
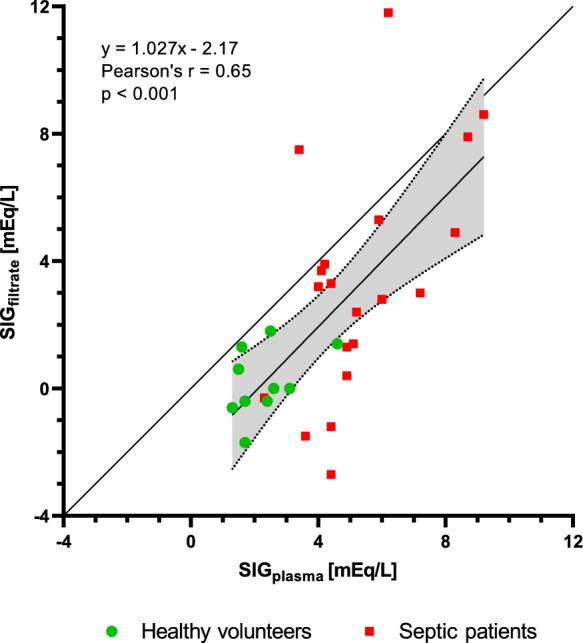
Experiment B. The relationship between the Strong ion gap in plasma (SIG_plasma_) and in protein-free serum filtrates (SIG_filtrate_) of healthy volunteers and septic patients. Linear regression line with 95% confidence bands and line of identity are shown

In Experiment B, the filtration process resulted in an almost complete elimination of protein, with TP and albumin concentrations below 0.4 g/L and 0.2 g/L, respectively. The strong ion gap in plasma (SIG_plasma_) in septic patients was significantly elevated (septic patients: 5.3 ± 1.8 mEq/L vs. healthy volunteers: 2.3 ± 1.0 mEq/L, p < 0.001) and the elevation persisted in protein-free serum filtrates (septic patients: 3.3 ± 3.7 mEq/L vs. healthy volunteers: 0.2 ± 1.1 mEq/L, p < 0.01). The difference between SIG_filtrate_ and SIG_plasma_ was similar in both groups (septic patients: − 2.0 ± 3.1 mEq/L vs. healthy volunteers: − 2.1 ± 1.1 mEq/L, p = 0.9). Individual data points are depicted in Fig. 3. For a detailed analysis of individual electrolytes, please see Table S1 in Supplementary Material.

## Discussion

### Experiment A: the pH-dependent charge of plasma proteins and phosphate

The primary aim of Experiment A was to externally validate pK_A_ and A_TOT_, originally determined by Staempfli and Constable [6], in a larger group of healthy volunteers, and to assess, whether the same pK_A_ is applicable to both healthy volunteers and critically ill patients.

**Fig. 4:**
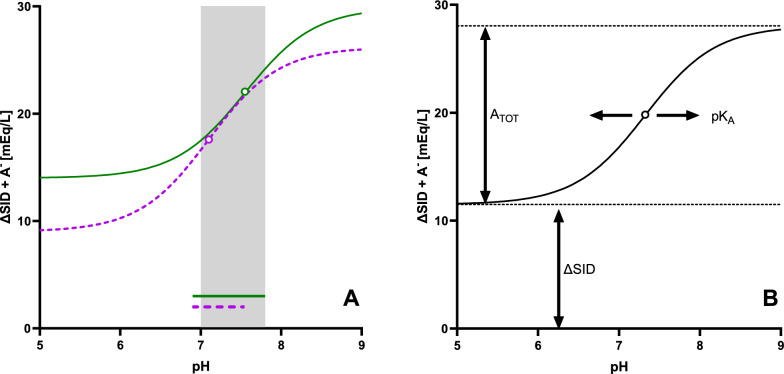
Panel **A**: Titration curves representing the net charge of plasma proteins and phosphate (ΔSID + A^−^) in healthy volunteers under the assumption of SID_unmeasured_ = 0. Solid green curve: our results (ΔSID = − 14.0 mEq/L, pK_A_ = 7.55, A_TOT_ = 15.9 mmol/L). Dashed purple curve: results of Staempfli and Constable (ΔSID = − 9.0 mEq/L, pK_A_ = 7.10, A_TOT_ = 17.2 mmol/L). The gray zone represents the pH range of 7.0 to 7.8. On each curve, a circle represents the pK_A_. The lines of appropriate color at the bottom represent the pH range explored in each study. Panel **B**: A diagram illustrating the impact of pK_A_, A_TOT_, and ΔSID on the position and shape of the titration curve of plasma proteins and phosphate under the assumption of SID_unmeasured_ = 0

In healthy volunteers, the estimated A_TOT_ and its equivalents expressed per gram of albumin or TP were not different from the values published by Staempfli and Constable [6]. Conversely, the estimates of pK_A_ and SID differed significantly (Fig. 1). We think that this discrepancy arises from different allocation of protein charge between the fixed and pH-dependent partitions. This is evidenced by our estimate of SID being approximately 4 mEq/L lower than that of Staempfli and Constable, despite identical SID_measured_ recorded in both studies (Fig. 1). To further support this theory, we advanced from statistical comparison of individual parameters, such as SID, pK_A_, and A_TOT_, to visual analysis of the curves representing the net protein and phosphate charges. We used ΔSID as a proxy for the fixed portion of their charge, assuming that SID_unmeasured_ is negligible in healthy volunteers (see Eqs. 1 and 5). As shown in Fig. 4A, the two titration curves overlap within the pH range of clinical interest, implying that both our parameters and those of Staempfli and Constable yield the same results when applied to healthy subjects. This agreement is possible despite significant differences in individual parameters because, within the relatively narrow pH range studied, a difference in pK_A_ (a lateral shift of the curve) can be compensated by an appropriate adjustment in ΔSID (its vertical position), as shown in Fig. 4B.

Both experimentally determined pK_A_ values (7.55 in this study and 7.10 [6]) are considerably higher than the average pK_A_ of the imidazole groups in histidine (6.75) [7]. This difference suggests that additional ionizable groups, such as the side chains of arginine and lysine or the α-amino terminus, contribute to plasma buffering.

Staempfli and Constable presented several pairs of pK_A_ and A_TOT_ values derived through different regression algorithms. One method used the average SID calculated from measured concentrations of strong ions [mean(SID_measured_)] as an input variable, estimating only pK_A_ and A_TOT_. In this approach, SID_unmeasured_ is assumed to be zero and plasma proteins and phosphate are treated as carrying no fixed charge, with their entire charge represented by [A^−^]. Applying this method to our data from healthy volunteers, we obtained results that closely matched those derived by Staempfli and Constable using the same approach (pK_A_: 6.63 ± 0.09 vs. 6.66 ± 0.12, p = 0.38; A_TOT_: 24.3 ± 1.8 vs. 23.3 ± 1.5, p = 0.16), further demonstrating strong agreement between the findings of both studies. The resulting titration curves are shown in Figure S4A in the Supplementary Material. The pK_A_ values obtained through this alternative regression algorithm are similar to those originally proposed by Stewart and later validated by Kowalchuk (6.70 [1] and 6.52 [2, 31]). Note that the assumption of no unmeasured strong ions limits the applicability of this method to healthy subjects.

Next, we investigated whether the same parameters could be used for acid–base diagnostics in healthy subjects and critically ill patients. We were particularly interested in sepsis, a condition characterized by elevated levels of unmeasured anions [13–18] and known alterations in the structure and function of plasma proteins [11]. To distinguish the influence of sepsis from critical illness in general, we also enrolled a group of patients without sepsis who were recovering from major surgery in ICU. Contrary to our hypothesis, the values of pK_A_ did not differ among the three groups. Not surprisingly, A_TOT_ was significantly lower in both groups of critically ill patients. However, its reduction was proportional to the degree of hypoproteinemia, resulting in a consistent A_TOT_/TP (but not A_TOT_/Alb) across all studied groups. These findings indicate that the behavior of pH-dependent charge of plasma proteins, i.e., their buffer properties, are not altered by critical illness. Despite that, TP concentration explains only a small portion of the variance in A_TOT_ (Pearson’s r = 0.37), indicating poor predictive value for determination of A_TOT_ in individual subjects (Fig. 2A).

The fact that A_TOT_/TP (rather than A_TOT_/Alb) remains stable in hypoproteinemia indicates that not only albumin, but also other plasma proteins possess buffer properties and contribute significantly to A_TOT_. Of note, in the Staempfli-Constable model of protein dissociation, A_TOT_ accounts for only part of the total charge carried by plasma proteins and phosphate, as their fixed charge makes a substantial contribution (Fig. 4B). This contrasts with the original Stewart model, where A_TOT_ along with pK_A_ determines the total charge of protein and phosphate at any given pH (Figure S4B in the Supplementary Material). In the Staempfli-Constable model, the main purpose of A_TOT_ is to determine the maximum slope of the titration curve at pH = pK_A_ (Fig. 4B) and it is, in fact, the only parameter affecting this property. By this logic, A_TOT_ serves a similar purpose as the noncarbonic buffer power employed in the classical linear model of protein dissociation [32]. Our preference of TP over albumin, therefore, aligns with authors who describe the protein buffer action in terms of TP [32, 33] rather than albumin alone [4, 5, 11]. Nevertheless, albumin and TP are strongly correlated and, in most patients, both parameters may be equally useful to estimate A_TOT_.

The final step in reproducing the analysis by Staempfli and Constable [6], was to update the equation for calculating net protein charge using the constants derived in this study. This yielded the following expression (for a detailed description of the methodology, see Text S2 in the Supplementary Material):

7 $$\left[{Pr}_{tot}^{-}\right]=TP (g/L)\times \left(0.108+\frac{0.230 \times TP \left(g/L\right)}{1+{10}^{7.55-pH}}\right)-Pi \left(mmol/L\right)\times \frac{1}{1+{10}^{6.8-pH}}$$

### Experiment B: the fixed charge of plasma proteins and phosphate

Having ruled out alterations in the pH-dependent charge of plasma proteins and phosphate in critical illness, we turned our focus to the fixed charge that these molecules carry. We defined ΔSID as a parameter comprising the fixed negative charge of plasma proteins and phosphate along with the charge of unmeasured strong ions (Eqs. 1 and 5). In healthy individuals, unmeasured ions only occur in negligible concentrations and, hence, the majority of ΔSID represents the fixed negative charge of plasma proteins. As both groups of the critically ill patients experienced a significant reduction in TP (by 33% in both groups) and albumin (by 33% and 50%, in postoperative and septic patients, respectively), their ΔSID clearly exceeded the expected level. Fixed phosphate charge (1 mEq/mmol of inorganic phosphorus) was not sufficient to explain this discrepancy, leaving only two possible causes: Either unmeasured strong anions (SID_unmeasured_) accumulate in plasma of critically ill patients, or critical illness alters the composition and/or structure of plasma proteins in a way that increases the amount of Pr^−^_fix_ per gram of TP.

To address this question we designed Experiment B, in which separate cohorts of healthy volunteers and critically ill patients with sepsis were enrolled. The goal was to use serum filtration to separate the charge carried by plasma proteins (Pr^−^_fix_ and majority of A^−^) from the negative charge of potentially present small (< 10 kDa) unmeasured anions (SID_unmeasured_) and determine in which of these two entities the unexplained charge persists. Based on this rationale, SIG_filtrate_ in septic patients had to match one of the following patterns: If, as per our hypothesis, plasma proteins carry additional negative charge, elimination of proteins would bring SIG_filtrate_ to the same level as observed in healthy volunteers. If, on the other hand, filterable unmeasured anions were present, SIG_filtrate_ in septic patients would remain elevated or may even rise slightly due to Gibbs-Donan effect. Our results clearly demonstrate that, in septic patients, SIG is elevated in both plasma and protein-free serum filtrate, proving that small unmeasured anions carry the unmeasured negative charge. This is further supported by the lack of a correlation between ΔSID and concentrations of TP or albumin in the combined dataset (Fig. 2C and D). However, when assessed separately within each of the three subgroups an anticipated trend becomes apparent (Figures S3C and D in the Supplementary Material).

### Strengths and limitations

Key strengths of our study are the use of modern equipment, advanced computational methods and a larger sample size compared to the original study by Staempfli and Constable, allowing for an external validation of their pioneer findings. The inclusion of critically ill patients, both postoperative and septic, makes our results generalizable to populations with severely altered composition of plasma proteins. Finally, extension of the study by Experiment B allowed us to assess not only the pH-dependent but also the fixed portion of protein and phosphate charge.

Our study has also several limitations. In *Experiment A*, the pH range achievable with pH tonometry is suboptimal to estimate pK_A_ and A_TOT_. Despite fully covering the clinically relevant spectrum, the recorded data points only describe the middle, nearly linear segment of the complex titration curve (Fig. 4A), which prevented us from determining which of the two currently available experimental estimates of pK_A_ better reflects protein dissociation. This also renders the regression model highly sensitive to data noise (e.g., measurement errors) and results in significant interindividual variability in the estimates of pK_A_, A_TOT_, and SID even in healthy volunteers. Furthermore, precision of point-of-care blood-gas analyzers is highest near physiological pH and PCO_2_ values, whereas the data points that most strongly influence the estimates of SID, pK_A_, and A_TOT_ are located at both extremes.

*In Experiment B*, several factors cause the concentration of individual chemical species in protein-free serum filtrates to differ from that in plasma. Most of these either affect healthy volunteers and septic patients equally (comparing measurements performed in heparinized blood and serum filtrates, measurement error at considerably elevated pH in serum filtrates) or do not alter SIG_filtrate_ at all (ongoing lactate production and CO_2_ elimination during sample preparation). The only exception is the Gibbs-Donan effect which may increase SIG_filtrate_ slightly. Extended analysis of these factors is presented in Text S3 in the Supplementary Material. Finally, the design of Experiment B did not allow for identification of specific substances responsible for the unmeasured charge.

## Conclusions

In this study, we experimentally determined the parameters required for the application of the Staempfli-Constable model of protein dissociation in clinical diagnostics. Although our estimates differ from those of Staempfli and Constable, the resulting titration curve is identical within the clinically relevant pH range. We demonstrated that the same values of pK_A_ (7.55) and A_TOT_/TP ratio (0.230 mmol/g) can be applied to both healthy volunteers and critically ill patients, including those with sepsis. Furthermore, we established that the unexplained negative charge often observed in plasma of septic patients is due to the presence of small unmeasured anions.

## Supplementary Information

Additional file 1.

## Data Availability

The complete datasets will be provided by corresponding author upon reasonable request.

## Abbreviations

Alb: Albumin
A_TOT_: Total concentration of dissociated and undissociated form of HA
CO_2_: Carbon dioxide
HA: Hypothetic weak nonvolatile acid that serves as a proxy for plasma proteins and phosphate in the Stewart model of acid–base equilibrium in plasma
ICU: Intensive care unit
K_A_: Acidic dissociation constant of HA
pK_A_: Negative decadic logarithm of K_A_
PCO_2_: Partial pressure of CO_2_
Phos^−^_fix_: Fixed charge carried by plasma phosphate
Pr^−^_fix_: Fixed charge carried by plasma proteins
SID: Strong ion difference—the net charge of all strong ions in plasma
SID_measured_: Net charge carried by the measured strong ions in plasma
SID_unmeasured_: Net charge carried by unmeasured strong ions in plasma
SIG: Strong Ion Gap
TP: Total protein
ΔSID: The difference between average SID_measured_ and estimated SID in each sample
