# Supplementary material for: Determination of pKA of nonvolatile weak acids in plasma of healthy volunteers and critically ill patients
Source: Intensive Care Med Exp. 2025 Jun 2;13:54. doi: 10.1186/s40635-025-00762-8 (PMC12130371; doi:10.1186/s40635-025-00762-8)
Supplement: Supplementary file 1 — Additional file 1. [file 40635_2025_762_MOESM1_ESM.pdf]

Supplementary Material

Determination of pK_A of Nonvolatile Weak Acids in Plasma of Healthy Volunteers and Critically Ill Patients

Martin Krbec, Serena Brusatori, Petr Waldauf, Alberto Zanella, Francesco Zadek, Victor van Bochove, František Duška, Thomas Langer, and Paul Elbers

Intensive Care Medicine Experimental

Correspondence to: František Duška, Department of Anaesthesia and Intensive Care Medicine, Third Faculty of Medicine, Charles University and FNKV University Hospital, Prague, Czech Republic, frantisek.duska@lf3.cuni.cz

Table of Contents

Supplementary Figure S1 2
Supplementary Figure S2 2
Supplementary Figure S3 3
Supplementary Figure S4 4
Supplementary Table S1 5
Supplementary Text S1 6
Supplementary Text S2 7
Supplementary Text S3 8
References 9

Supplementary Figure S1

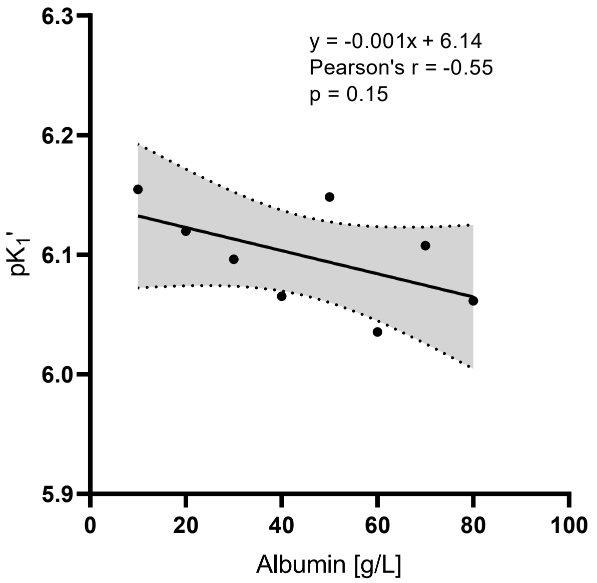

The relationship between albumin concentration and experimentally determined pK_1' in artificially prepared solutions with varying protein content. A regression line with 95% confidence bands is shown.

Supplementary Figure S2

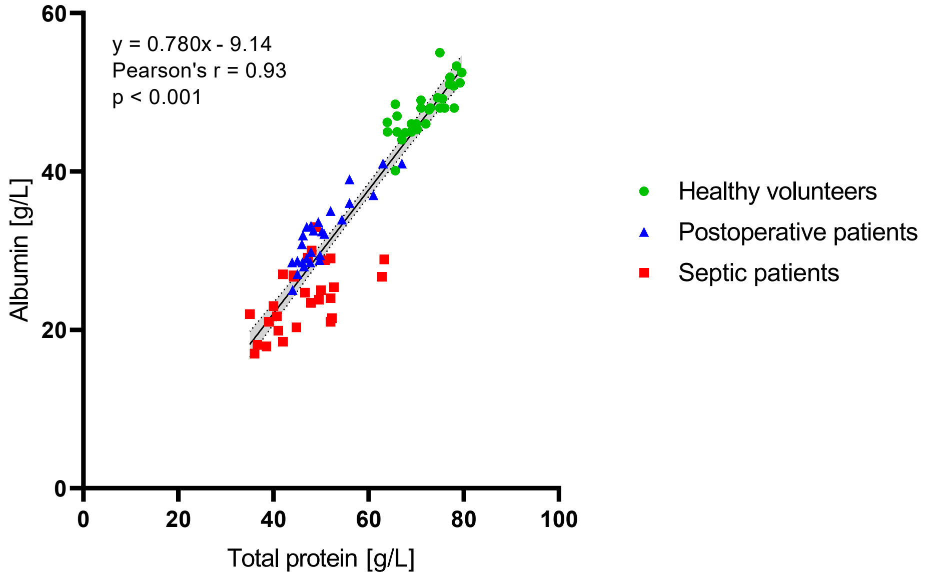

Experiment A.

The correlation between albumin and total protein concentration. A regression line with 95% confidence bands is shown.

Supplementary Figure S3

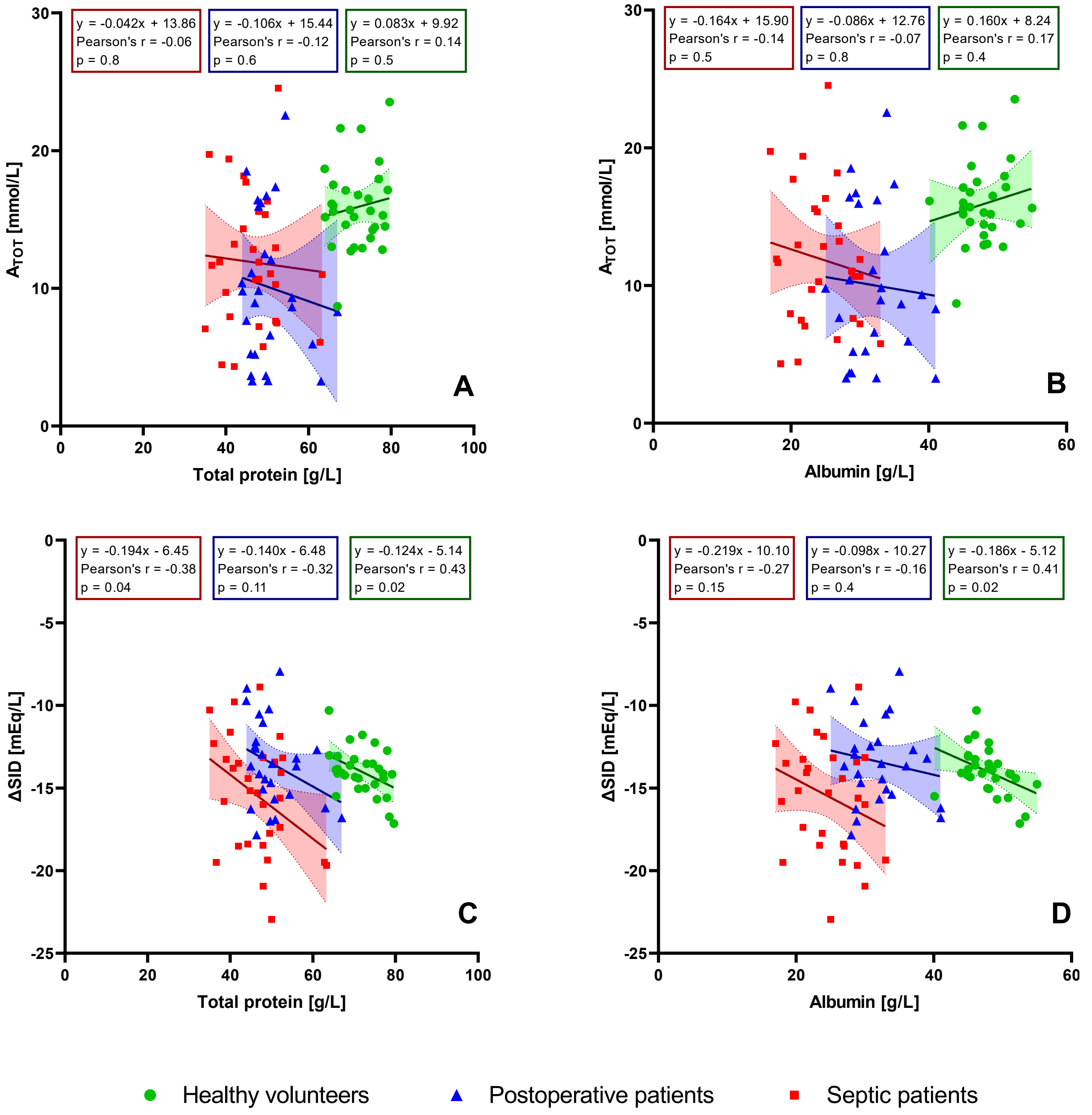

Experiment A.

Panels A and B: the relationship between the estimated A_{TOT} and total protein or albumin concentration within each studied group.

Panels C and D: the relationship between ΔSID (i.e., the fixed charge of plasma proteins and phosphate, and unmeasured strong ions) and total protein or albumin concentration within each studied group.

Linear regression line with 95% confidence bands is shown in all graphs.

Supplementary Figure S4

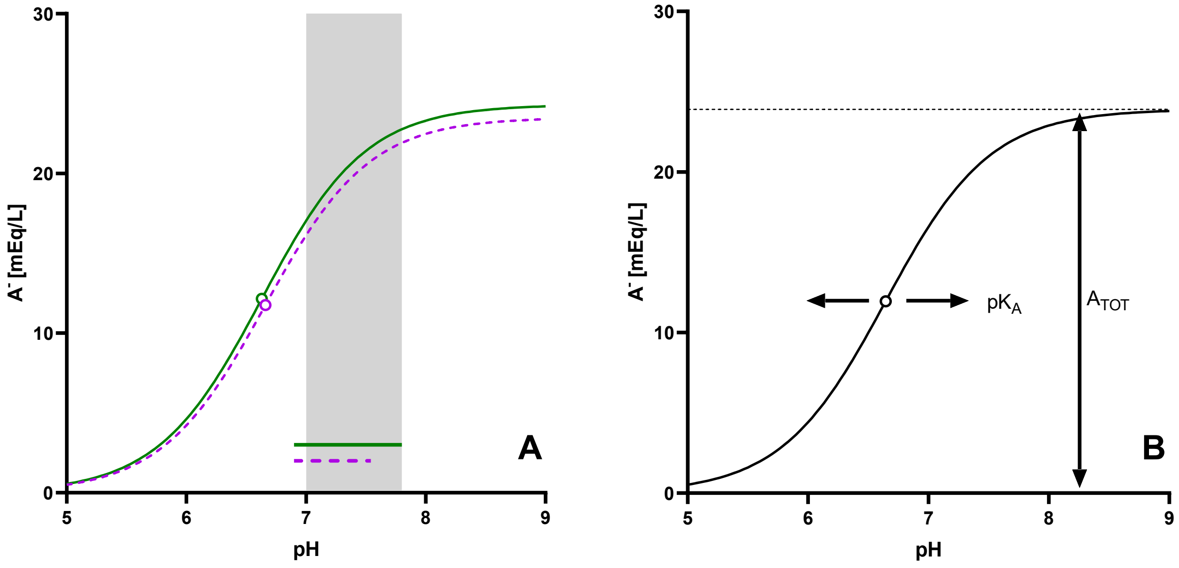

Experiment A.

Panel A: titration curves representing the net charge of plasma proteins and phosphate (A^-) in healthy volunteers derived with the alternative regression algorithm (using measured SID as an input variable, assuming absence of unmeasured ions and no fixed charge of plasma proteins and phosphate). Solid green curve: our results (pK_A = 6.63, A_{TOT} = 24.3 mmol/L). Dashed purple curve: results of Staempfli and Constable (pK_A = 6.66, A_{TOT} = 23.3 mmol/L). The grey zone represents the pH range of 7.0 to 7.8. On each curve, a circle represents the pK_A. The lines of appropriate colour at the bottom represent the pH range explored in each study.

Panel B: A diagram illustrating the impact of pK_A and A_{TOT} on the position and shape of the titration curve of plasma proteins and phosphate derived with the alternative regression algorithm.

Supplementary Table S1

	Healthy volunteers			Septic patients		
	Plasma	Serum filtrate	p value	Plasma	Serum filtrate	p value
pH	7.34 ± 0.04	7.87 ± 0.03	<0.001	7.36 ± 0.12	7.81 ± 0.10	<0.001
PCO_2, mmHg	55 ± 9	17 ± 1	<0.001	44 ± 8	17 ± 2	<0.001
$[HCO_3^-]$, mmol/L	29.2 ± 2.1	31.1 ± 1.8	<0.001	25.2 ± 5.5	27.4 ± 6.4	<0.001
$[Na^+]$, mmol/L	144 ± 2	136 ± 1	<0.001	141 ± 7	136 ± 7	<0.001
$[K^+]$, mmol/L	4.3 ± 0.4	4.1 ± 0.3	0.04	4.5 ± 0.8	4.3 ± 0.8	0.01
$[Ca^{2+}]$, mmol/L	1.25 ± 0.02	1.08 ± 0.03	<0.001	1.09 ± 0.14	1.01 ± 0.13	<0.001
$[Cl^-]$, mmol/L	104 ± 2	107 ± 2	<0.001	106 ± 5	108 ± 6	0.001
$[Lac^-]$, mmol/L	1.5 ± 0.8	2.5 ± 0.7	<0.001	2.3 ± 2.5	2.9 ± 2.5	<0.001
Magnesium, mmol/L	0.82 ± 0.04	0.51 ± 0.02	<0.001	0.90 ± 0.15	0.73 ± 0.15	<0.001
Phosphate, mmol/L	1.2 ± 0.2	1.2 ± 0.2	0.001	1.5 ± 0.9	1.4 ± 0.9	0.21

Experiment B.

Comparison of pH, PCO_2 and electrolyte composition of plasma and protein-free serum filtrates of healthy volunteers and septic patients. The p value refers to paired t-test.

Supplementary Text S1

The Value of pK_1' in Protein-Poor Fluids

The apparent first dissociation constant of carbonic acid in biological solutions (pK_1') is, despite its name, not constant. It varies with factors such as temperature [1, 2], ionic strength [1, 3], and, according to some authors, pH [2, 4]. Recent guidelines [5] recommend the fixed values of 6.095 and 6.105 as appropriate for measurements performed in blood and isolated plasma, respectively. For protein-free biological fluids, however, a consensus has not been established. For instance, pK_1' in the cerebrospinal fluid has been reported within a relatively wide range of 6.110 to 6.147 [4]. This raised the question of whether the value used for plasma (6.105) can also be applied to the protein-free filtrates of serum analysed in Experiment B of this study. To investigate this, we conducted a brief supplementary experiment in which pK_1' was measured.

Using commercially available human serum albumin (A9511, Sigma-Aldrich, Darmstadt, Germany), normal saline, sodium hydroxide, and water, we prepared eight solutions with protein concentration ranging from 10 to 90 g/L and ionic strength of 303 ± 6 mmol/L. The determination of pK1' was conducted using a method developed by Siggaard-Andersen [1]. In summary, two samples were prepared from each albumin solution by diluting it with either water (sample A) or hydrochloric acid (sample B), achieving a [Cl^-] difference of exactly 10 mmol/L. Both samples underwent CO_2 tonometry, with pH and PCO_2 measured repeatedly using a standard blood gas analyser (ABL90, Radiometer, Copenhagen, Denmark). At least 10 data points were recorded for each sample, from which the PCO_2 corresponding to a pH of 7.4 was determined by polynomial interpolation. Calculation of pK_1' was based on the principle that, at any given pH, $[HCO_3^-]_B$ must be 10 mmol/L higher than $[HCO_3^-]_A$. Applying this relationship at the pH of 7.4 and combining the Henderson-Hasselbalch equation for the samples A and B provides:

Eq. S1: $$pK_1' = 7.4 + log\left[0.0307 \times \frac{(PCO_2)_A - (PCO_2)_B}{10}\right]$$

The mean pK_1' in our solutions was 6.099 ± 0.040, which is not different from 6.105 ($p = 0.68$). No correlation was found between protein content and pK_1' (Pearson's $r = -0.56$, $p = 0.15$, Figure S1 in this Supplementary Material), implying that the same pK_1' can be used to calculate [HCO_3^-] in plasma as well as protein-poor fluids.

Supplementary Text S2

The Equation for Net Protein Charge

In theory, quantification of pH-independent and pH-dependent protein charge is possible using CO_2 tonometry data, if the concentration of protein is varied while $SID_{unmeasured}$ remains constant. The range of variation in albumin and TP concentrations observed in healthy volunteers is too narrow for a reliable analysis, as also noted by Staempfli and Constable [6]. Pooled data from all participants show substantially broader variation in albumin and TP concentrations. However, the heterogeneity in the amount of unmeasured strong anions, as revealed in Experiment B, precludes the accurate estimation of fixed protein charge.

Given the above limitations, we were compelled to rely on estimates of total protein charge at normal pH reported by other authors: $[Pr^{-}_{tot}]_{7.4}$ = 0.179 mEq/g of TP [7].This amount of charge may be divided into the fixed and pH dependent fractions:

Eq. S2: $$[Pr^{-}_{tot}]_{7.4} = \left[Pr^{-}_{fix}\right] + \left[Pr^{-}_{pH-dep}\right]_{7.4}$$

The pH-dependent partition may be obtained from $[A^-]$ by subtracting the pH-dependent charge of phosphate:

Eq. S3: $$[Pr^{-}_{tot}]_{7.4} = \left[Pr^{-}_{fix}\right] + [A^{-}]_{7.4} - \left[Phos^{-}_{pH-dep}\right]_{7.4}$$

With the above expression for net protein charge, Stewart's expression for $[A^-]$, and an explicit quantification of pH-dependent phosphate charge, Eq. S3 can be rewritten as:

Eq. S4: $$\left[Pr^{-}_{fix}\right] = 0.179 \times TP(g/L) - \frac{A_{TOT}}{1+10^{pK_A-7.4}} + \frac{Pi\ (mmol/L)}{1+10^{6.8-7.4}}$$

By inserting the measured TP and Pi concentrations, along with individual estimates of pK_A and A_{TOT} of the subjects enrolled in Experiment A, we obtained $[Pr^{-}_{fix}]$ of 6.0 ± 2.1 mEq/L and $[Pr^{-}_{fix}]$/TP ratio of 0.108 ± 0.033 mEq/g. Using this ratio, together with Stewart's expression for $[A^-]$, the mean values of pK_A and A_{TOT}/TP derived in this study, and an explicit quantification of pH-dependent phosphate charge, Eq. S3 can be generalized for any pH:

Eq. S5: $$[Pr^{-}_{tot}] = 0.108 \times TP\ (g/L) + \frac{0.230 \times TP\ (g/L)}{1+10^{7.55-pH}} - \frac{Pi\ (mmol/L)}{1+10^{6.8-pH}}$$

Given that (1) the titration curves representing the net charge of plasma proteins and phosphate in healthy volunteers (Figure 4A) agreed closely between the two studies, (2) the pK_A and A_{TOT}/TP derived in this study did not significantly differ between groups, and (3) the same source for net negative charge of plasma proteins at normal pH was used, it follows that our Eq. S5 and Eq. 9 from the study by Staempfli and Constable [6] will yield close results within the clinically relevant pH range.

Supplementary Text S3

The Gibbs-Donan Effect and Other Factors Affecting the Electrolyte Composition of Serum Filtrates

The electrolyte composition of plasma and protein-free serum filtrates differs significantly (Supplemental table S1). There are several factors responsible, but only some of them affect $SIG_{filtrate}$ and its relationship to SIG_{plasma}.

First, the method of anticoagulation differs. Electrolyte measurements for SIG_{plasma} were performed in blood anticoagulated by electrolyte-balanced heparin, while for $SIG_{filtrate}$ serum was obtained from blood collected in clot activator tubes, possibly causing a small discrepancy due to dilution error. Importantly, this error affects healthy volunteers and septic patients equally and is, in our opinion, the principal factor behind the ~2 mEq/L difference between SIG_{plasma} and $SIG_{filtrate}$ in both populations. Note that the well-known discrepancy between point-of-care testing of anticoagulated blood and central laboratory measurements in serum [8, 9], explained by the use of direct vs. indirect ion-selective electrodes [10], does not apply in this case as the principal electrolytes were in both cases measured by a point-of-care analyser, i.e., using direct ion-selective electrodes.

Second, the pH, PCO_2, and lactate values used in the calculation of SIG_{plasma} reflect their in-vivo levels but are considerably altered in serum filtrates due to ongoing red cell metabolism and continuous escape of CO_2 into the atmosphere. While this makes the values obtained in serum filtrates clinically irrelevant, there is no effect on the resulting $SIG_{filtrate}$. The lactic acid produced is immediately buffered, ensuring that the rise in lactate is matched by an equivalent reduction in the negative charge of available buffer anions (bicarbonate, phosphate). Similarly, CO_2 loss and the resulting reduction in bicarbonate are offset by increased ionization of the remaining buffer species (phosphate).

Finally, the Gibbs-Donnan effect applies during the filtration process, reducing the concentrations of cations (Na^+, K^+, Ca^{2+}, and Mg^{2+}) and increasing the concentrations of low molecular weight anions (Cl^-, HCO_3^-, and Lac^-) in serum filtrates. This process produces a protein-free electroneutral fluid, in which the estimation of unmeasured ions may be performed without the hypothesized confounding effect of plasma proteins. Whether the Gibbs-Donan effect also affects $SIG_{filtrate}$ depends on which chemical species are responsible for its elevation. If, as per our hypothesis, serum proteins are responsible for the elevation of SIG_{plasma} in septic patients, $SIG_{filtrate}$ would be similar to that in healthy volunteers (close to zero) and unaffected by the Gibbs-Donan effect. If, on the other hand, small filterable anions are responsible for the elevation of SIG_{plasma} in sepsis, $SIG_{filtrate}$ would not only remain elevated but may even rise slightly, akin to $[Cl^-]$.

References

1. Siggaard-Andersen O (1962) The first dissociation exponent of carbonic acid as a function of pH. Scand J Clin Lab Investig 14:587–597. https://doi.org/10.1080/00365516209051289

2. Severinghaus JW, Stupfel M, Bradley AF (1956) Variations of Serum Carbonic Acid pK' With pH and Temperature. J Appl Physiol 9:197–200. https://doi.org/10.1152/jappl.1956.9.2.197

3. Tibi L, Bhattacharya SS, Flear CTG (1982) Variability in pK′1 of human plasma. Clin Chim Acta 121:15–31. https://doi.org/10.1016/0009-8981(82)90206-6

4. Mitchell RA, Herbert DA, Carman CT (1965) Acid-base constants and temperature coefficients for cerebrospinal fluid. J Appl Physiol 20:27–30. https://doi.org/10.1152/jappl.1965.20.1.27

5. CLSI (2009) Blood Gas and pH Analysis and Related Measurements (CLSI document C46-A2), 2nd ed. Clinical and Laboratory Standards Institute, Wayne, PA

6. Staempfli HR, Constable PD (2003) Experimental determination of net protein charge and Atot and Ka of nonvolatile buffers in human plasma. J Appl Physiol 95:620–630. https://doi.org/10.1152/japplphysiol.00100.2003

7. Van Leeuwen AM (1964) Chapter IX: Summing-up. Acta Med Scand Suppl. 422:191–202. https://doi.org/10.1111/j.0954-6820.1964.tb05680.x

8. Morimatsu H, Rocktäschel J, Bellomo R, et al (2003) Comparison of Point-of-Care Versus Central Laboratory Measurement of Electrolyte Concentrations on Calculations of the Anion Gap and the Strong Ion Difference. Anesthesiology 98:1077–1084. https://doi.org/10.1097/00000542-200305000-00009

9. Chacko B, Peter JV, Patole S, et al (2011) Electrolytes assessed by point-of-care testing - Are the values comparable with results obtained from the central laboratory? Indian J Crit Care Med 15:24–29. https://doi.org/10.4103/0972-5229.78219

10. Holbek CC (2002) Understanding the different values in electrolyte measurements. In: Acutecaretesting.org. https://acutecaretesting.org/-/media/acutecaretesting/files/pdf/understanding-the-different-values-in-electrolyte-measurements.pdf
